# Assessment of dynamics and variability of organic substances in river bank filtration for prioritisation in analytical workflows

**DOI:** 10.1007/s11356-024-34783-9

**Published:** 2024-08-27

**Authors:** Sebastian Handl, Kaan Georg Kutlucinar, Roza Allabashi, Christina Troyer, Ernest Mayr, Reinhard Perfler, Stephan Hann

**Affiliations:** 1 Department of Water, Atmosphere and Environment, Institute of Sanitary Engineering and Water Pollution Control, University of Natural Resources and Life Sciences, Vienna (BOKU), Muthgasse 18, 1190 Vienna, Austria; 2 Department of Chemistry, Institute of Analytical Chemistry, University of Natural Resources and Life Sciences, Vienna (BOKU), Muthgasse 18, 1190 Vienna, Austria

**Keywords:** River bank filtration, Non-targeted analysis, LC-HRMS, Organic micropollutants, Exposure

## Abstract

Bank filtration supports the growing global demand for drinking water amidst concerns over organic micropollutants (OMPs). Efforts to investigate, regulate and manage OMPs have intensified due to their documented impacts on ecosystems and human health. Non-targeted analysis (NTA) is critical for addressing the challenge of numerous OMPs. While identification in NTA typically prioritises compounds based on properties like toxicity, considering substance quantity, occurrence frequency and exposure duration is essential for comprehensive risk management. A prioritisation scheme, drawing from intensive sampling and NTA of bank filtrate, is presented and reveals significant variability in OMP occurrence. Quasi-omnipresent substances, though only 7% of compounds, accounted for 44% of cumulative detections. Moderately common substances, constituting 31% of compounds, accounted for 50% of cumulative detections. Rare compounds, comprising 61%, contributed only 6% to cumulative detections. The application of suspect screening for 31 substances to the dataset yielded results akin to NTA, underscoring NTA’s value. Correlation between both methods demonstrates the efficacy of high-resolution mass spectrometry-based NTA in assessing temporal and quantitative OMP dynamics.

**Supplementary Information:**

The online version contains supplementary material available at 10.1007/s11356-024-34783-9.

## Introduction

Water infiltration from lakes and rivers to the groundwater is a natural process. Its capacity can be enhanced by increasing the hydraulic gradient via the operation of wells in the proximity of the surface water bank (Hiscock and Grischek 2002). This technique is called river bank filtration (RBF) and represents a significant, efficient and cost-effective method for purifying surface water. The technique involves filtration, adsorption, biodegradation and mixing with native water and affects suspended solids, bacteria, viruses or parasites, as well as inorganic and organic substances (OS) (Masse-Dufresne et al. 2019; Munz et al. 2019). Due to the reduced extent of necessary treatment steps, RBF is considered a vital method to meet the increasing water demand worldwide (Hu et al. 2016; Rossetto et al. 2020; Wahaab et al. 2019). Uhl et al. (2022) argue that declining groundwater tables due to climate change result in an increasing number of rivers or river sections changing from gaining (exfiltrating groundwater) to losing (infiltrating river water) conditions. Combined with the increasing concentration and diversity of substances in the river water, this significantly threatens groundwater quality in general and RBF sites in particular.

Organic substances include organic micropollutants (OMPs), which range from pharmaceuticals, endocrine active substances, pesticides and disinfection by-products (Richardson and Kimura 2020) to industrial substances such as permanent cationic contaminants (Köppe et al. 2023) or per- and poly-fluoroalkyl substances (Herkert et al. 2020). Even though often only detected in low concentrations (ng L^−1^), these substances are still of increasing concern due to their properties, such as environmental persistence or adverse health effects and toxicity (Berrou et al. 2021; Kondor et al. 2020). The number of industrial and natural chemical compounds ranges in the thousands and is increasing every year (Hollender et al. 2019; Kondor et al. 2023).

Richardson and Kimura (2020) describe mass spectrometry in combination with different separation techniques as the standard analytical method to detect and quantify OMPs in environmental samples. High-resolution mass spectrometry (HRMS), in combination with liquid chromatography (LC), is capable of detecting especially polar and moderately polar substances (Kutlucinar et al. 2022). Non-targeted analysis (NTA) describes a strategy for LC-HRMS that, in contrast to targeted or suspect screening strategies (profiling), covers all unidentified/unknown compounds which are amenable under the selected analytical and data processing conditions (Gosetti et al. 2016). Besides identification confidence level 1, which demands for authentic standard substances, the maximum confidence level achievable based on single-stage HRMS is level 4. Based on MS/MS derived fragment information, on the other side, level 2 can be reached (Schymanski et al. 2014). Based on a non-targeted approach, Köppe et al. (2023) showed that a range of persistent cationic molecules with significant health concerns increased in the sediment of the rivers Rhine and Saar in Germany. Moreover, it has been shown that OMPs are subject to biotransformation processes in the environment, which results in transformation products of different toxicity (Eysseric et al. 2022). Therefore, the capability of NTA to consider unexpected substances like transformation products is particularly valuable in studies concerning the environmental fate of OMPs (Yang et al. 2023).

Due to the high number of compounds detected by NTA in environmental samples and the exhaustive efforts necessary to confirm them, many studies aimed to develop tools for the prioritisation of the substances for the subsequent identification process. These efforts range from establishing mass spectral databases to developing algorithms based on different statistical concepts to support retention time and retention time index prediction (Yang et al. 2023).

Exposure is a crucial factor for assessing the adverse health effects of OMPs (Xue et al. 2021). Great efforts have been made to assess the exposure of humans and other organisms to a vastly increasing number of chemical substances (Zhao et al. 2021). Exposure assessment demands for measuring or estimating the amount, frequency, duration and route of exposure to a chemical. Further, potential sources of the compounds need to be identified and assessed in terms of the likelihood of contact and contact time (i.e. acute vs chronic exposure). Based on these findings, it is evaluated whether the predicted exposure levels are within acceptable ranges.

In this context, the variability and dynamics of OMPs in RBF are essential input parameters to consider when investigating the environmental and human health-related impact of these substances. Further, the dynamics of OMPs should also be considered when prioritising efforts for the identification of compounds from NTA. This investigation builds upon our previous work (Kutlucinar et al 2022) regarding analytical methodology for NTA. In contrast to that work, which focused on the remediation and elimination of OMPs in river bank filtration between the river and the groundwater, here we present different aspects of substance dynamics considering temporal and semi-quantitative variability based on HRMS signals, drawn from a comprehensive study of bank filtrate. The developed prioritisation categorisation is demonstrated based on 31 selected OMPs with very low detection limits and concentrations in the environmental samples from a river bank filtration site at the river Danube in Vienna (Austria). This set of compounds was extracted by application of suspect screening/post-target screening (Gosetti et al. 2016) to the raw dataset from NTA.

## Material and methods

### Study site

The study site is located along the river Danube in the north of Vienna (Austria). The Danube’s catchment area up to this location is above 100.000 km^2^, and its medium discharge is 1910 m^3^/s. The six bank filtrate wells (BFWs) form a wellfield to abstract bank filtrate along the left bank of the Danube (Fig. S1). The wells have a distance of 130 m to the left river bank. The strong water level gradient along the left river bank towards the northeast ensures a continuous groundwater flow also during pumping interruptions. Furthermore, the intrusion of native groundwater into the wellfield can be excluded. This circumstance predestines the study site to investigate phenomena concerning water quality in river bank filtration.

The aquifer consists of sandy, well-rounded carbonate gravel. Its bottom is situated between 141.86 and 153.36 m a.s.l. with an average of 149.72 m a.s.l. The bottom of the Danube riverbed lies at about 154.77 m a.s.l. (median). The resulting average aquifer thickness below the Danube is 5.05 m. The median water level in the Danube is 161.35 m a.s.l.

### Field sampling

Mixed-bank-filtration (MBF) samples, each representing a mix of all six BFWs, were taken from a single sampling tap in the collection pipe that transports water from all six BFWs. The sampling was conducted 5 days a week for 8 months, from February to October 2018. It was interrupted by three short periods of operational shutdown of the wellfield between May and July. In total, 143 samples were collected. The comprehensive investigation program ensured adequate coverage of the dynamics. Further, the full range of meteorological conditions was covered during the selected period with the influence of snow melt, lowest and highest temperatures of the year and summer high waters, according to Fig. S2. The observed high waters during the investigation had a maximum discharge of 3760 m^3^/s, which is well below the 1-year high water discharge of 5290 m^3^/s. The total abstraction rate from the wellfield ranged from 70 to 330 L/s, with abstraction rates from individual wells ranging from 10 to 100 L/s.

### Blanks, reference and quality control samples

Blank samples (field blanks) were taken weekly by filling ultra-pure water in sampling bottles. Reference samples were produced by spiking ultra-pure water with 31 selected substances (Table S1) at a concentration of 0.1 µg/L. The substance list includes pesticides to account for agricultural production in the catchment area, medical products and tracer substances (caffeine, saccharin), which account for treated wastewater. Further sample preparation of blank and reference samples corresponded to that applied for field samples.

For each of the three measurement batches and each ionisation mode, pooled quality control (QC) samples were prepared by blending 3 or 4 field samples, which were randomly selected from the corresponding sample batch. Technical replicates of these QC samples were measured throughout the batch (replicates from the same HPLC vial).

### Analytical method

#### Sample preparation

Sample preparation and conservation was performed according to Kutlucinar et al. (2022). For sampling of river water, sample glass flasks (500 mL) were used. The MBF samples were filtered within 11 h after sampling using a 0.45-µm cellulose filter, aliquoted in 38-mL aluminium vessels (plastic screw cap with Teflon inlet) and stored until sample preparation at − 20 °C. All flasks (sampling and storage) were washed using demineralised water and the laboratory washing agents ProCare Lab 10 MA and ProCare Lab 30 C (Miele) in a laboratory dishwasher (Miele, Wals, Austria) and rinsed twice with ultrapure water. Further, the cleaned bottles were heated at 180 °C for 16 h.

#### Data acquisition with HPLC-TOFMS

Non-targeted analysis using the HPLC-TOFMS platform along with data processing followed the procedures outlined in Kutlucinar et al. (2022) with one modification (60 mL instead of 18 mL sample volume was used). The HPLC-TOFMS consisted of a 1290 Infinity II LC system from Agilent Technologies, Santa Clara, USA and a time-of-flight mass spectrometer (Agilent 6230 B TOFMS) featuring an Agilent Jet Stream interface for electrospray ionisation. The chromatography system was equipped with a pre-column and a separation column (Waters—ACQUITY UPLC® HSS T3, 1.8 µm particle size, 2.1 mm I.D. * 150 mm length). Prior to analysis, analyte enrichment was performed via solid phase extraction using a Gilson GX-271 ASPEC® (Middleton, USA) and 60 mg 3 cc Oasis HLB cartridges as described by Kutlucinar et al. (2022).

In general, our NTA method aimed at high coverage of organic substances, as both, the solid phase material (HLB) and the chromatographic stationary phase (fully wettable C18 material) provide a wide polarity range from ionic to non-polar molecules.

#### Feature extraction and processing for non-targeted workflow

Feature extraction and peak picking were performed by applying the batch-recursive molecular feature extraction workflow (MassHunter Profinder, Agilent Technologies). Intra-sample alignment of ion features to molecular features, composed of at least two ions, was conducted, and a feature exclusion was performed based on the feature extraction score. Following the molecular recursive feature extraction, compounds detected in a single sample with a score above 75 (rMFE target score) were retained in the compound list. Results from the automatic chromatographic peak integration were validated visually. The rMFE target score is a weighted value considering the deviation of aligned individual features over the measured samples from the calculated median values in terms of retention time, accurate mass and isotopologue abundances.

In the case of NTA, only relative quantification could be applied. Therefore, trueness of quantification could not be assessed. However, an intercomparison with the results of the suspect screening has been performed (see “Suspect screening” section). The precision of the NTA method was assessed via the pooled QCs.

#### Feature extraction and processing for suspect screening

For targeted extraction of spiked compounds, feature extraction was performed with MassHunter Workstation Quantitative Analysis for TOF version B10.1 (Agilent Technologies), according to Kutlucinar and Hann (2021). After chromatogram extraction based on retention times and m/z values ([M + H]^+^) for each compound, the extracted chromatograms were controlled visually for correct peak integration and, if necessary, manually reintegrated. Compounds with mass accuracy > 10 ppm or signal-to-noise ratio (S/N) < 3 were considered outliers and excluded. The method was validated in terms of trueness and precision via standard additions to selected groundwater samples in our study comparing SPE with direct injection analysis (Kutlucinar and Hann 2021).

#### Intensity normalisation

Intensity correction was based on all features present in each QC sample from non-targeted feature extraction. First, an instrument sensitivity drift correction was applied for each of the three batches separately. The intensities of the features were tested for a linear trend along the measurement sequence. The trend was considered significant if the median of the *p *-value over all regression models was below 0.05. In these cases, drift correction for all intensities considered the median slope derived from the QC samples. Second, inter-batch effects were evaluated and corrected. The median intensity within each batch, the median intensity over all batches and the ratio between these variables were calculated for each feature. For each batch, the median of the variable ratio over all features gives the inter-batch correction factor. Consequently, the drift-corrected intensities from the first normalisation step were multiplied by the inter-batch-correction factor. The application of the normalisation procedure resulted in a significant reduction of intensity variability as presented in Fig. S3 and Table S2.

#### Assessment of sample validity

Sample validity evaluation is based on each sample’s intensity of internal standards (IS). For positive ionisation mode, D_5_-labelled atrazine and for negative ionisation mode ααα-trifluoro-m-toluic acid was considered, and feature extraction was performed according to the suspect screening workflow. After normalisation of intensities as described above, the median intensity of the IS was calculated for each batch. A total of 24 samples (17 positive and 7 negative) were excluded from the evaluation since the intensity of the internal standard was below 70% or above 120% of the internal standard’s median batch intensity.

#### Artefact and background elimination for non-targeted screening

In addition to the filters used within the MassHunter Profinder workflow for artefact elimination, a three-step artefact identification method was employed. This method involved grouping compounds based on similarities in mass and retention time, marking putative pairs of compounds (redundant or split hits), calculating the relative congruence of occurrence for each pair and applying a linear regression model to the intensity measurements from each pair of compounds across all samples. Compounds were considered likely artefacts or redundant hits if the relative congruence of occurrence exceeded 85% and the *p* value of the linear regression fell below 0.05. Among pairs or clusters of probable artefacts, the compounds with lower median abundances were deemed artefacts and removed from the dataset.

Background elimination was executed based on the peak area abundance (ion volumes representing the sum of all ions of a specific compound) with four exclusion criteria. First, all entities with an intensity below 1000 counts per second (cps) were removed. Second, all compounds with a maximum abundance across all samples below 5000 cps were removed. Third, to account for possible contamination (e.g. plasticisers, per- and poly-fluoroalkyl substances, detergents) during sample collection and preparation, compounds detected in field blanks with an intensity above 1000 cps were excluded. Forth, LOQ (limit of quantification) values were computed separately based on the corresponding SPE blanks and instrumental blanks. Entities of compounds were eliminated if their highest signal fell below the highest LOQ value from the corresponding blanks (Kutlucinar et al. 2022).

### Evaluation of organic substance dynamics

The dynamics and variability of OS are described by their temporal and quantitative dynamics. The temporal dynamics can be evaluated through (i) the frequency of their occurrence over the whole investigation period, (ii) the average duration between detections and (iii) the recurrence dynamics. The quantitative dynamics expresses how the concentration of a substance varies between their occurrences.

#### Temporal dynamics

The number of samples in which a particular compound was detected indicates the absolute frequency of occurrence. The relative frequency is calculated by relating the absolute frequency to the total number of samples. The compounds are categorised into three groups depending on the frequency of their occurrence, according to Table S3.

The average duration between detections (ADBD) is calculated by ordering the dates of all the samples in which a particular compound was detected. Further, the duration between consecutive detections is calculated. For each compound, the mean is calculated and rounded. This metric can only be determined for compounds detected at least twice.

The recurrence dynamics describe how the presence of compounds in the samples changes over time. The detection of a compound can be classified into four groups based on its presence in previous samples, according to Table S4. Group A represents compounds that were detected in the immediately preceding sample. Compounds that were not detected in the last sample but were present at some point during the week prior to the current sample form group B. Group C represents compounds that were not detected in the previous week. Finally, compounds detected for the first time in the entire investigation period form group D.

#### Quantitative dynamics

In addition to the concentration of a substance in the sample, the physicochemical properties of the substance strongly influence the substance-specific sensitivity obtained by ESI-HRMS. Therefore, a direct comparison of intensities (ion volumes) of different substances is not meaningful. However, intensities can be used to compare the relative quantity of a particular substance in different samples.

The variability in the intensity of a substance can be described by the deviation of the intensity in each sample from the mean value across all samples (Formula S1). The standard deviation of the intensity of the compounds across all samples is used as a measure. It is calculated according to Formula S2. Thus, for each compound, the maximum difference from the mean can be expressed by a multiple of the standard deviation, allowing substances to be compared with each other in terms of their variability in relative intensity.

## Results and discussion

### Number and occurrence of compounds (NTA)

A total of 5923 compounds were detected across all samples. Using positive ionisation, 4431 compounds were detected, while using negative ionisation 1492 were detected. Seven hundred seventy-six compounds (494 in positive, 282 in negative mode) were only detected in non-environmental samples (reference samples, quality control samples or field blanks) but never detected in any environmental sample. A total of 4537 compounds (3518 in positive, 1019 in negative mode) were either detected in field blanks or the highest measured intensity was below the limit of quantification calculated from instrumental and SPE blank samples and consequently were excluded. Only 610 compounds (419 in positive, 191 in negative mode) showed an intensity above the limit of quantification in at least one environmental sample. Five compounds were detected in both ionisation modes, as shown in Table S5.

The time series depicting the number of detected compounds (Fig. S4) shows that each sample had at least 70, on average 96, and up to 156 different compounds. Using negative ionisation, 3 to 44 compounds per sample (average 14) were detected, while using positive ionisation 60 to 128 compounds (average 82) were detected. On certain dates (e.g. early September for positive ionisation mode), noticeably more compounds were detected. No linear trend or seasonality was observed. Oberleitner et al. (2020) reported similar ratios between maximum and minimum number of detected compounds per sample at Ems River sites (148% and 195%) but found higher variability at Ruhr River sites (154% and 630%). In accordance with this study, there was no apparent correlation with hydrological boundary conditions.

### Temporal dynamics of compounds (NTA)

#### Recurrence dynamics

**Fig. 1:**
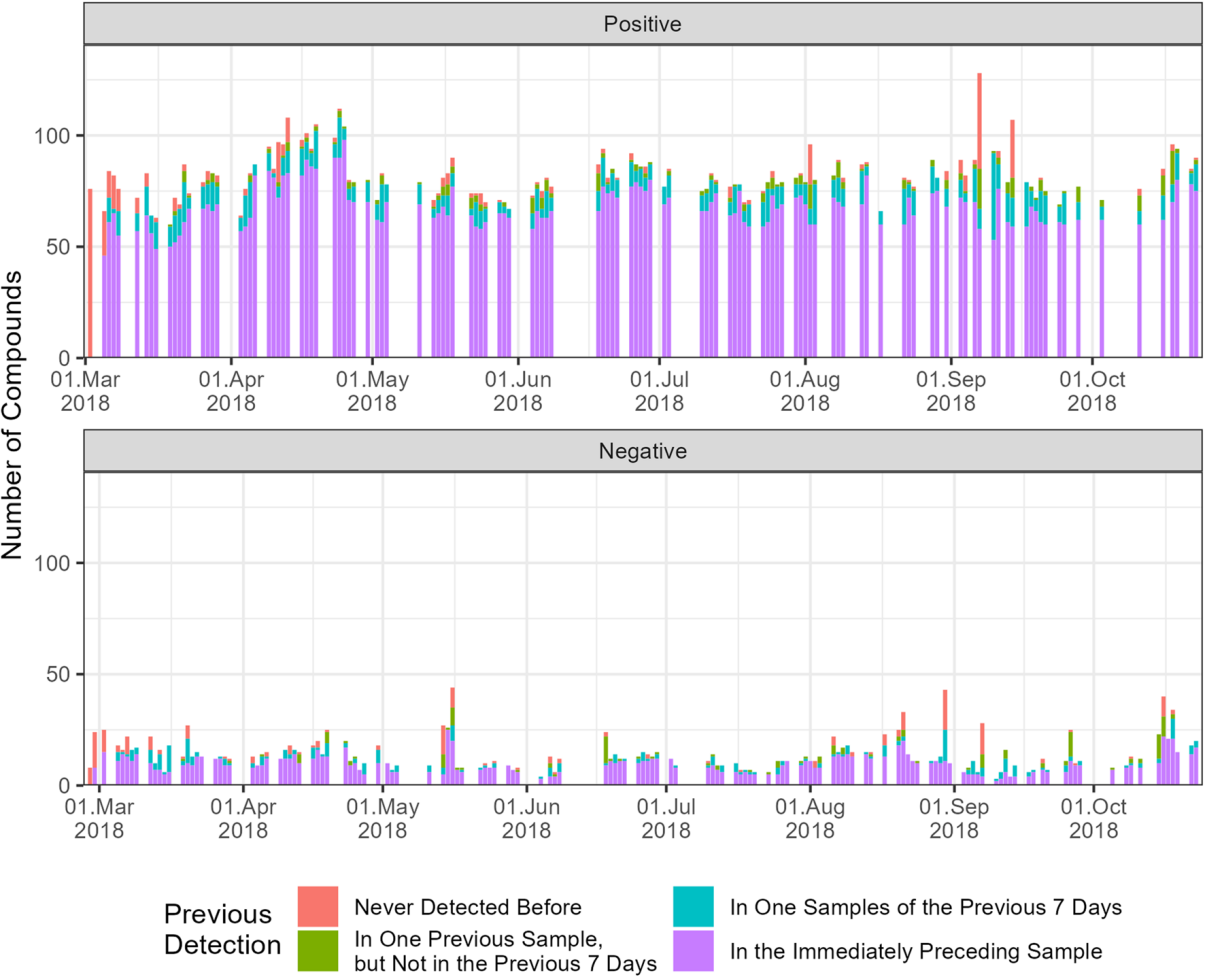
Time series of the number of detected compounds according to their recurrence dynamics (colours) for positive (top) and negative (bottom) ionisation modes

The temporal recurrence dynamics of the valid compounds are illustrated in Fig. 1. On average (and median), 79% (83%) of compounds were also detected in the immediately preceding sample (purple), 12% (10%) were detected at least once in the previous week (turquoise), 5% (3%) were detected earlier but not in the previous week (green), and 8% (3%) were detected for the first time (red). Consequently, the initial sample has all compounds marked red. Notably, also samples with more compounds than average comprise a higher number of compounds detected for the first time (marked in red).

The high similarity to the previous day’s sample (and less than 10% difference from last week’s samples) indicates a very high short-term uniformity.

#### Frequency of occurrence

**Fig. 2:**
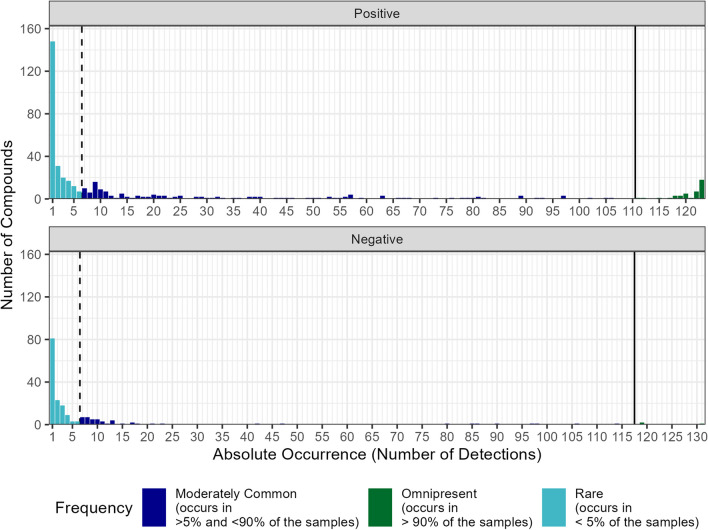
Histogram of absolute frequency of occurrence for positive (top) and negative (bottom) ionisation mode. The relative frequency is indicated by colour

Figure 2 displays all 610 compounds (positive mode 419, negative mode 191) according to their absolute frequency of occurrence for both ionisation modes. Two hundred twenty-six compounds (37%, 148 positive, 78 negative) occur only once throughout the study, represented by the bars furthest to the left in the diagrams. For the analysis with positive ionisation, 19 compounds (3.1%) are present in all valid samples (total 123 times) as indicated by green bar furthest to the right in the upper diagram.

We analysed all 610 compounds, dividing them into three groups based on their relative frequency of occurrence. The black lines indicate the frequency limits. Compounds found in less than 5% of samples are located on the left of the dashed line. Those found in more than 90% of samples are to the right of the solid line.

The largest group accounts for 61% of compounds (375 total, 235 positive, 140 negative), and each was found in less than 5% of samples. Omnipresent compounds, numbering 44, were present in more than 90% of samples, making up 7% of the total number of compounds. The remaining 191 compounds (31%) categorised “moderately common” were neither frequently nor rarely detected. From a water protection viewpoint, these compounds are of particular interest since they are detected often enough to represent a potential risk but do not occur often enough to be covered in regular quality assessment with sufficient certainty. Kondor et al. (2020) investigated the frequency of occurrence of pharmaceutically active compounds and report similar values for the three occurrence classes in bank filtration sites in Hungary. Of the targeted 111 substances, 32 were detected in at least one of the bank filtrate samples. Sixty-three percent of the substances were detected rarely, many of these in concentrations below 1 ng/L. Thirty-four percent of the 32 substances would classify as moderately common, and 3% of the substances were found to be omnipresent in the samples. Compounds that occur omnipresent in bank filtrate samples might refer to substances that are not only ubiquitous present in the river water but are also persistent during the bank filtration process. Moreover, the release of natural organic compounds like humic substances from the soil is also possible. Richardson and Ternes (2022) state that the increasing use of pharmaceuticals and hormones results in their ubiquitous presence in surface waters. Further, per- and poly-fluoroalkyl substances qualify as possible substances with ubiquitous presence due to their high persistence in the environment. The rare occurrence of substances in bank filtrate might result from their rare occurrence in the river water. Another example is substances that occur in the river constantly but penetrate the river bank filtration system only rarely due to either unusually high concentrations or temporarily occurring conditions that reduce or hinder their usual degradation.

**Fig. 3:**
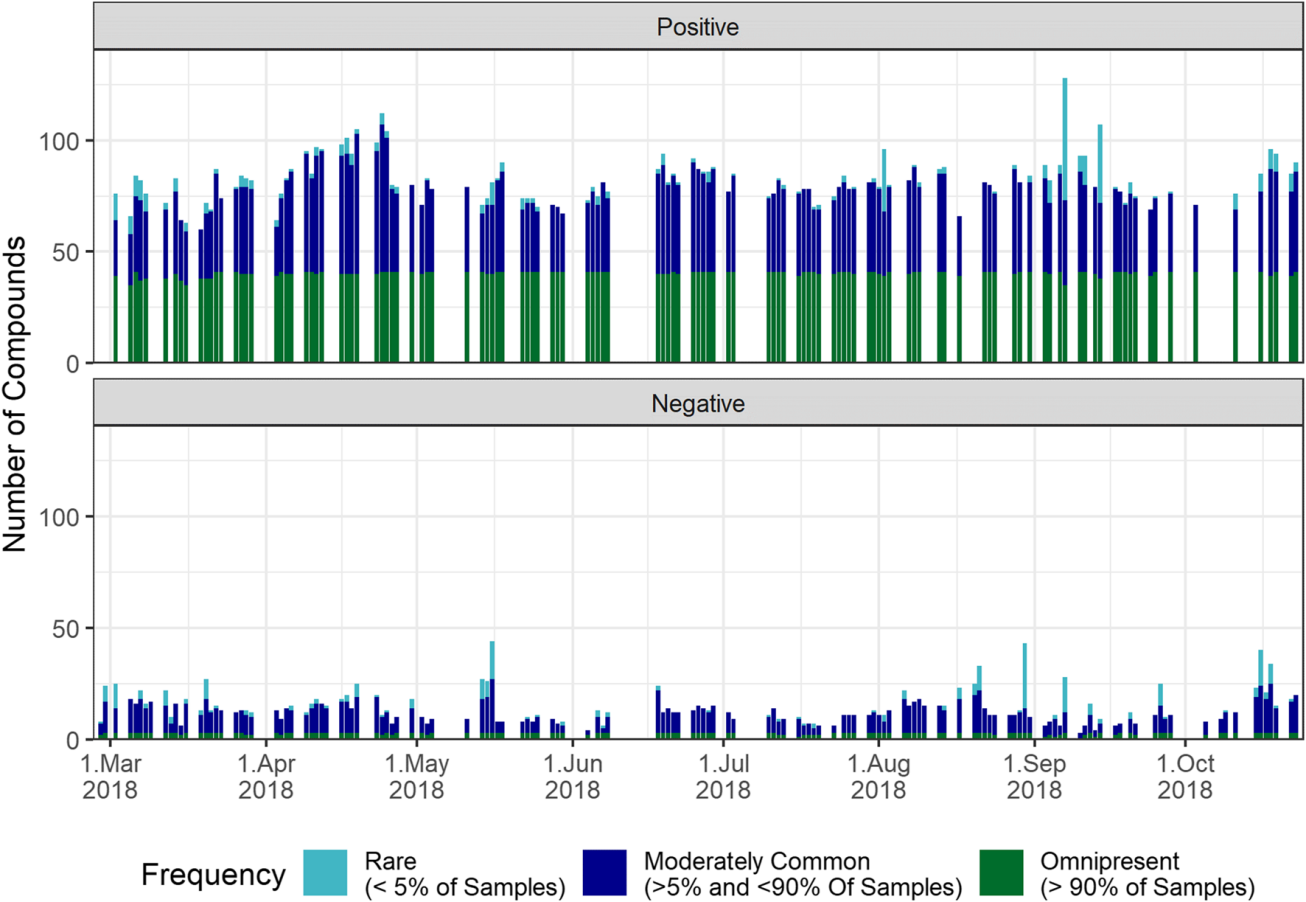
Time series of the number of compounds according to their frequency of occurrence class for positive (top) and negative (bottom) ionisation mode. Relative frequency is indicated by colour

Figure 3 displays the time series of environmental samples, with features differentiated by their frequency group by distinct colours. The green group (omnipresent) is observed to be consistently present throughout the investigation, with an average of 40 (positive mode) and 3 (negative mode) compounds per sample. This group accounts for approximately 44% of cumulative detections (entirety of every instance a substance is detected, including multiple detections). The turquoise group, representing rare compounds, only accounts for a small portion (around 6%) of the cumulative detections and is notably present in samples containing an unusually high number of compounds. The dark blue group (moderately common) accounts for 50% of the cumulative detections and seems responsible for the medium-term fluctuations observed in the time series.

**Fig. 4:**
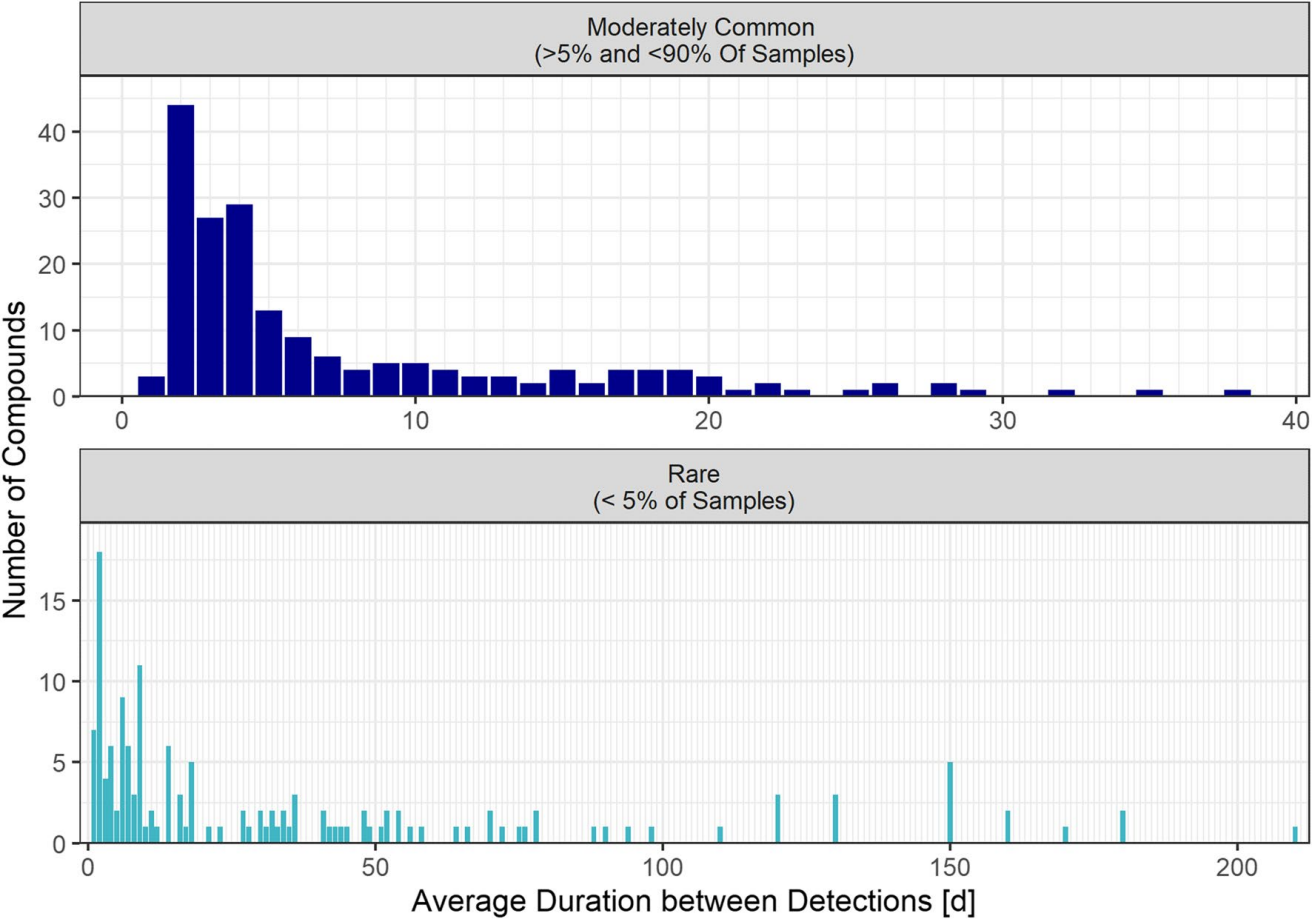
Number of compounds by average duration between detections for different abundance groups (moderately common: top; rare: bottom). Omnipresent compounds are not displayed. The average duration between detections is 2 days for all compounds in this group

Figure 4 displays the distribution of the compounds according to the average duration between detections, separated for the three frequency classes. The values range from 1 to 210 days for rare compounds and are widely dispersed. However, most compounds in this group have an average temporal distance between detections of less than 25 days. The average duration between detections of compounds in the moderately common group ranges from one to 38 days, with a mean of 7.5 days for all compounds in the group. All compounds in the omnipresent group have a value of 2 days. This is because sampling was not carried out on weekends and holidays, and the values for each compound were rounded.

For moderately common compounds, the comparison of number of detections with the average duration between detections in Fig. S5 shows that substances with low ADBD (< 3) and low frequency (< 10 samples), thus exhibiting a plume-like temporally concentrated occurrence, account for a considerable share of 11% of this substance group.

### Quantitative dynamics of compounds (NTA)

**Fig. 5:**
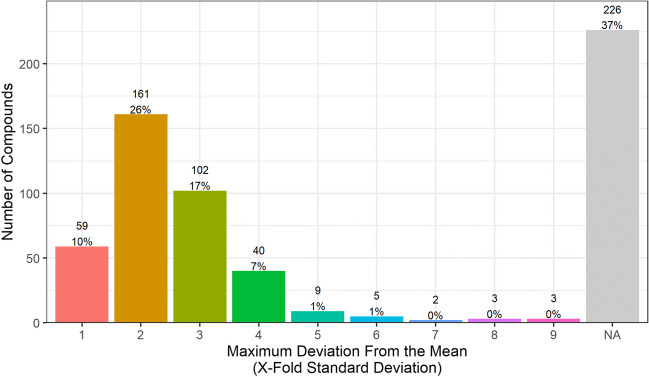
Number of compounds by semi-quantitative variability of compound intensities (maximum deviation from the mean expressed in standard deviations). Compounds only detected once cannot be evaluated and are categorised “NA”

Figure 5 presents the distribution of compounds based on their maximum intensity variance (for both positive and negative ionisation mode). The number of compounds in each category and their corresponding percentage of the total compounds are indicated above the bars. The largest portion of compounds, i.e. 37% (226), was detected only once throughout the study, and as a result, no standard deviation could be calculated for them. These are grouped under “NA” (not available). The group of compounds with moderate intensity variances (up to three times the standard deviation) accounts for 53% (322) of the total compounds, including 10% (59) with a onefold, 26% (161) with a twofold and 17% (102) with a threefold standard deviation. Sixty-two compounds (10%) exhibit a pronounced variance in intensity, exceeding the threefold standard deviation for the entire study period.

Using a similar approach, Albergamo et al. (2019) showed, based on the variability of intensities obtained with NTA in a wellfield with a travel time of up to 60 years, that for a large number of compounds the highest abundance occurred during the 1990s. Köppe et al. (2023) found linear trends of quaternary ammonium compounds based on the intensity variance from NTA.

Figure S6 displays the time series of the variance in intensity over the study period. Samples with unusually high numbers of detected compounds show a high proportion of compounds for which no standard deviation of the intensity can be calculated. These are compounds that were detected only once in the entire study period. Table S6 shows the average share of each group over all samples. Most detected compounds (44.6% per sample) have a maximum deviation from the mean of 3 SD (standard deviation). The second largest group with 4 SD accounts for 25.9% of detections per sample, followed by the group of 2 SD with an average share of 12.5%. Only 2.3% show a maximum variance of the mean of 1 SD. All other groups account, on average, for 17.5% of the detection in a sample.

### Suspect screening

Out of the 31 evaluated substances, the intensities of the 19 substances (2.4-D,alachlor, atenolol, bentazon, chloridazon, clothianidin, dichlorprop, diuron, isoproturon, linuron, MCPA, mecoprop, metazachlor, metobromuron, monolinuron, pethoxamid, propazine, propiconazole, thiamethoxam) were below the detection limit in all samples. This is in accordance with JDS4 (ICPDR 2021), which showed that alachlor is absent and isoproturon and diuron were only found in 3 of 51 surface water samples along the Danube in 2021. For metazachlor, the same authors reported an occurrence in 69% of Danube samples. Data from Dragon et al. (2019) shows a 19% occurrence of metazachlor and 47% of isoproturon in river bank filtration in Poland. Nagy-Kovács et al. (2018) report the detection of diuron in the Danube but not in the river bank filtration samples in Hungary.

**Fig. 6:**
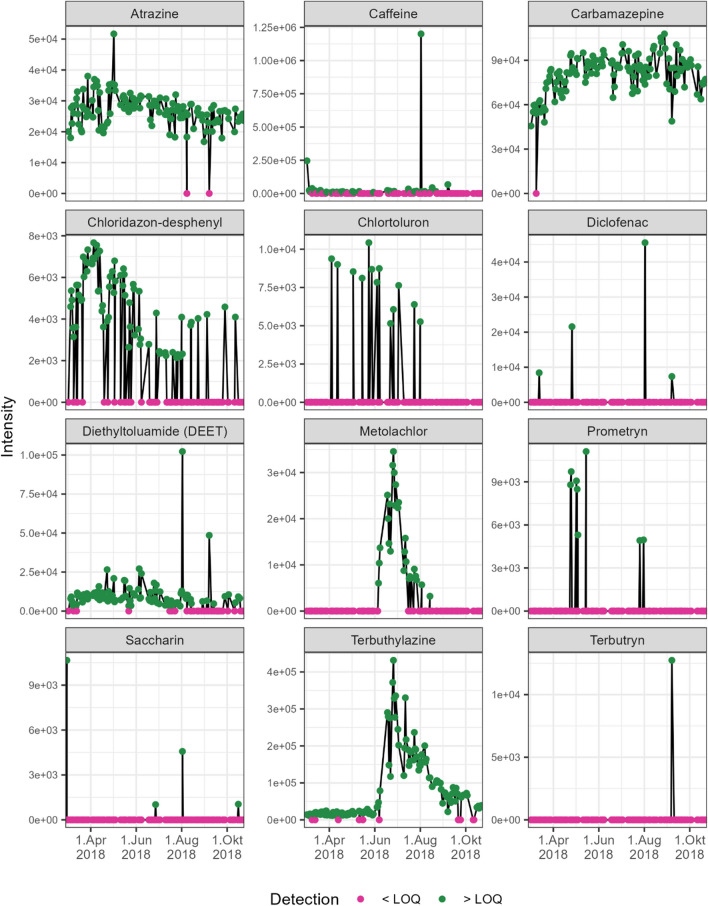
Time series of intensities of substances detected above the detection limit. The measurement values in the environmental samples are shown as green dots connected by a line. Measurements that were below the detection limit are represented as pink dots at the intensity of zero

Figure 6 shows the time series of the remaining 12 substances. For these substances, the parameters for temporal dynamics (ADBD and relative frequency) as well as quantitative dynamics (maximum deviation from the mean) were calculated (Table S7). Figure S7 shows the time series in the context of intensities in the reference samples (1 µg/L). Three substances (caffeine, diclofenac and saccharin) were detected at least once above the reference samples’ intensity, indicating concentrations above 0.1 µg/L. Kondor et al. (2020) report for diclofenac, a similarly low frequency of occurrence (1.1%) but maximum concentrations of only 0.0016 µg/L from river bank filtration in Budapest (Hungary). Their results contrast with the findings of the present study in terms of caffeine behaviour, showing a low occurrence frequency and a low maximum concentration of 0.022 µg/L. The intensities of the remaining nine substances were above the detection limit in at least one MBF sample. Still, at the same time, these were significantly below the intensities of the reference samples, representing the current limit of 0.1 µg/L.

Atrazine, carbamazepine and terbuthylazine are categorised as omnipresent substances. All three substances, therefore, show an ADBD value of 2 days. Of these three substances, atrazine shows the highest (5 SD) quantitative dynamics resulting from one single measurement in April. JDS4 showed that atrazine and terbuthylazine were present in all, and carbamazepine was found in 47 of the total 51 Danube samples taken in 13 countries in 2019. The maximum concentrations during JDS4 ranged from 0.01 µg/L (atrazine) and 0.058 µg/L (carbamazepine) to 0.087 µg/L (terbuthylazine) in Danube samples. In bank filtration samples, the authors report the presence of atrazine in all 7 collected samples (ICPDR 2021). There is a noticeable increase in terbuthylazine in the middle of June, which then continuously decreases to the initial level. The elevated quantitative dynamics (maximum deviation from the mean of 4 SD) results from this plume-like appearance. Carbamazepine shows moderate intensity variations (3 SD). There are no significant time dependencies noticeable for atrazine and carbamazepine. Carbamazepine is generally reported to have high persistence under oxic conditions (Yamamoto et al. 2009). Kondor et al. (2020) also report a high frequency of occurrence (> 99%) and a moderate maximum concentration of 0.5 µg/L for carbamazepine in bank filtration along the Danube.

DEET also shows frequencies above 70%, resulting in an ADBD value of 2 days. One single outlier results in the high quantitative dynamics of DEET (8 SD).

Caffeine, chloridazon-desphenyl and metolachlor are detected in more than 10% of the samples. ADBD for all substances is between 3 and 4 days. The lower relative frequency combined with a lower ADBD for metolachlor compared to caffeine indicates a concentrated appearance. The high quantitative dynamics of caffeine (maximum deviation from the mean of 6 SD) results from a single outlier. In less than 4% of the samples, a concentration of caffeine above 0.1 µg/L was detected.

The herbicides metolachlor and terbuthylazine are used among others in maize cultivation, explaining the plume-like increase in spring. JDS4 (ICPDR 2021) report the presence of metolachlor in all 51 Danube samples in 2021. Chlortoluron and prometryn show a relative frequency of 5–10%. Both substances also show increased ADBD values above 10 days and low quantitative dynamics with a maximum deviation from the mean of 2 SD. Dragon et al. (2019) reported higher detection frequencies at a river bank filtration site in Krajkowo (Poland), with 38% for prometryn and 76% for chlorotoluron with detection limits of 5 ng/L.

Diclofenac and saccharin are categorised as rare substances. In combination with a non-concentrated appearance, this results in high ADBD values. Both substances also show low quantitative dynamics. Munz et al. (2019) report good attenuation for diclofenac under oxic conditions but low attenuation up to persistent behaviour under low oxygen concentrations. Zeeshan et al. (2023) report a high degradation of diclofenac and saccharin that correlated with residence time from column experiments aiming at simulating river bank filtration under oxic conditions. Terbutryn is only detected once. Therefore, neither ADBD nor quantitative dynamics can be calculated. This is also in accordance with JDS4 (ICPDR 2021), which reported terbutryn in only 3 of 51 samples along the Danube in 2021. Nagy-Kovács et al. (2018) report the detection of terbutryn in the Danube but not or only in very low concentrations in bank filtration.

### Comparison of results from suspect screening and NTA

Intensities and results of eight compounds from suspect screening (SSC) were compared to those from the NTA workflow, i.e. signals with the same m/z and retention time. Based on this assignment, the detection/intensities of the substances obtained by the two different methods were compared in all field samples and their repetitions were assessed as “valid” based on the internal standard evaluation. As shown in Table S8, samples were grouped into four classes: (A) detection in NTA and no detection in SSC, (B) no detection in NTA and detection in SSC, (C) no detection in neither method and (D) detection with both methods. Classes A and B comprise the diverging classes, and C and D comprise the converging classes. The ratio of converging samples ranged from 73.5% (chlortoluron) to 99.3% (carbamazepine), with an average of 90.9%. This result confirms a good accordance between the two methods concerning substance detection.

**Fig. 7:**
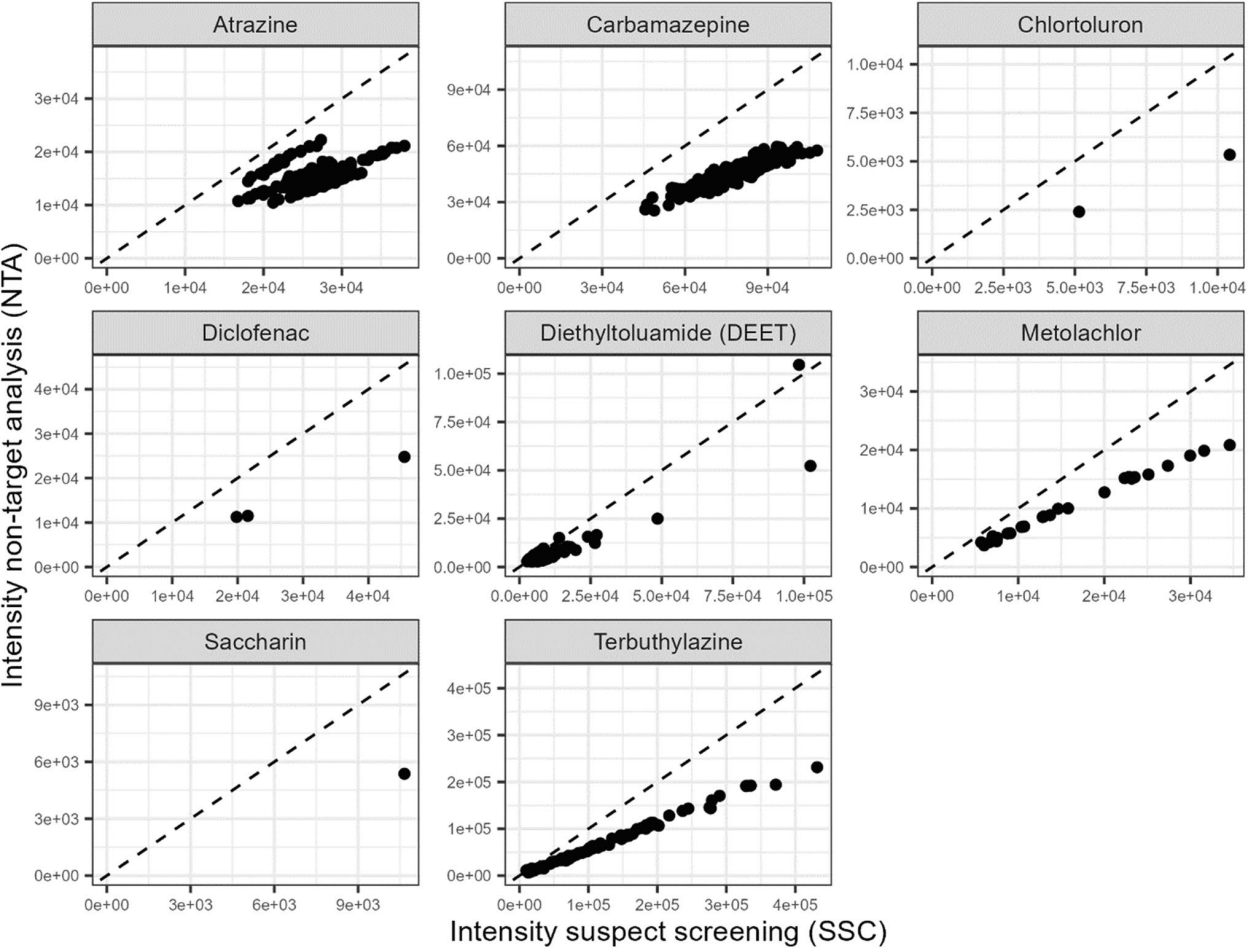
Scatter plot of intensities obtained from SSC and intensities from NTA for field samples and repetitions

Figure 7 compares intensities for all samples in detection class D. A close correlation between the measurements from both methods is evident for all eight substances. This is especially pronounced for carbamazepine, chlortoluron, metolachlor and terbuthylazine. Generally, higher intensity results are obtained from the suspect screening method than the NTA method. This can be explained by the different isolation windows of the extraction of the chromatographic signals from the spectral data obtained, as the peak extraction for NTA was performed via MassHunter Profinder and the peak extraction for SSC was obtained from MassHunter Quantitative Analysis.

### Insights and strategies for the prioritisation of substances for identification

Based on the findings regarding the described parameters, the following six considerations can be applied to strategically prioritise compounds for identification.

1. Generally, the rarer a substance occurs, the smaller is the potential impact. Therefore, rare compounds and especially those detected only once are considered very low priority.
2. With increasing frequency, also the priority increases. We suggest considering moderately common compounds with high priority.
3. Regarding compounds with similar frequency, the priority of those which occur more concentrated, expressed by short ADBD, is higher, since a concentrated plume-like occurrence is also associated with higher impact. Compounds with these characteristics might also qualify for particular interest in studies regarding source tracking.
4. Generally, (quasi-) omnipresent substances have a very high priority.
5. The existence of comprehensive targeted monitoring programs can also influence the prioritisation strategy. If a wide range of substances and their potential transformation products are analysed regularly and are not detected, the probability that (quasi-) omnipresent compounds are natural organic substances increases.
6. Compounds with high maximum deviation from the mean that occur relatively frequent indicate substances with a low LOQ and low baseline concentration but considerable peak concentrations. The time series of caffeine (Fig. 6) shows an example for this occurrence.

## Conclusion

The dynamics of organic substances in bank filtration can be investigated in terms of temporal and quantitative aspects. Regarding temporal dynamics, three concepts (1) the frequency of occurrence, (2) average duration between detections (ADBD) and (3) recurrence dynamics were developed and evaluated. Quantitative dynamics were evaluated based on the maximum variance of intensities. These concepts were assessed based on the comprehensive investigation of 143 samples collected within a period of 9 months from a bank filtration site in Vienna.

On average, about 96 compounds were detected per sample. The statistical analysis of the occurrence showed a large variability between the substances. The quasi-omnipresent substances represent only 7% of the compounds but account for nearly half (44%) of the cumulative detections. Moderately common substances represent 31% of the compounds and account for 50% of the cumulative detections. The remaining 61% of compounds rarely occur and therefore only account for only 6% of the cumulative detections.

The evaluation of compound recurrence shows that, on average, nearly 80% of compounds from a sample can be expected to be also detected in a sample from the directly preceding day. Further, it revealed that samples being in the high range regarding the number of detected substances also had particularly many substances that were never detected before and also belonged to the rarely occurring substances.

The presented criteria for temporal dynamics (ADBD and relative frequency and recurrence dynamics) are suitable to describe the occurrence dynamics of qualitative parameters. The combination of ADBD and relative frequency allows distinguishing between characteristic occurrence patterns like regular and concentrated (plume) presence in time.

The metric for quantitative change (maximum deviation from the mean) is very sensitive to single outliers. A possible solution to make this metric more stable might be to use quantiles (e.g. 95% quantile) instead of maximum deviation from the mean.

Thirty-one substances were identified and quantified in the suspect screening approach. The statistical evaluation showed that the temporal distribution can strongly differ between substances. Carbamazepine and atrazine showed continuous detection and a low variability in concentration. Terbuthylazine and metolachlor showed increasing followed by decreasing concentrations over time associated with a contamination plume. Other substances like diclofenac showed up only occasionally in seemingly unrelated incidents.

Only three substances (caffeine, diclofenac and saccharin) were detected with intensities above the intensities in the reference samples, indicating a concentration higher than 0.1 µg/L. All these exceedances occurred in a low number of samples.

SSC and NTA showed very similar results concerning detection in the field samples and their repetitions, which indicates the validity of the NTA workflow.

In general, NTA of mass spectrometry data is a valuable method for the evaluation of temporal and quantitative dynamics of organic substances, as exposure is a crucial concept for the evaluation of health effects of chemical substances. The main factors for exposure are the substance concentration and the duration of the contact. In this sense, the presented investigation contributes to an objective evaluation of risk from organic micropollutants in drinking water. Further topics in exposure assessment not covered in this investigation are threshold effects (adverse health effects only detectable for exposures above a certain threshold) or bioavailability (the portion of the substance that reaches the bloodstream or target organs). These are chemical-specific aspects demanding for the identification of the detected compounds, which can be obtained by fragmentation-based mass spectrometric strategies and dedicated mass spectrometric databases.

## Supplementary Information

Below is the link to the electronic supplementary material.

Supplementary file 1 (DOCX 1.15 MB)

## Data Availability

The authors declare that the data supporting the findings of this study are available within the paper and its Supplementary information files. Should any raw data files be needed in another format they are available from the corresponding author upon reasonable request.
