# Supplementary material for: Assessment of dynamics and variability of organic substances in river bank filtration for prioritisation in analytical workflows
Source: Environ Sci Pollut Res Int. 2024 Aug 27;31(40):53410–23. doi: 10.1007/s11356-024-34783-9 (PMC11379727; doi:10.1007/s11356-024-34783-9)
Supplement: Supplementary file 1 — (DOCX 1.15 MB) [file 11356_2024_34783_MOESM1_ESM.docx]

**Supplementary information**

Assessment of dynamics and variability of organic substances in river bank filtration for prioritisation of identification in analytical workflows

Environmental Science and Pollution Research

Sebastian **Handl**^1^*, Kaan Georg **Kutlucinar**^1,2^, Roza **Allabashi**^1^, Christina **Troyer**^2^, Ernest **Mayr**^1^, Reinhard **Perfler**^1^, Stephan **Hann**^2^

^1^ University of Natural Resources and Life Sciences, Vienna (BOKU), Department of Water, Atmosphere and Environment, Institute of Sanitary Engineering and Water Pollution Control, Muthgasse 18, 1190 Vienna, Austria

^2^ University of Natural Resources and Life Sciences, Vienna (BOKU), Department of Chemistry, Institute of Analytical Chemistry, Muthgasse 18, 1190 Vienna, Austria

**corresponding author:* [*sebastian.handl@boku.ac.at*](mailto:sebastian.handl@boku.ac.at)

*
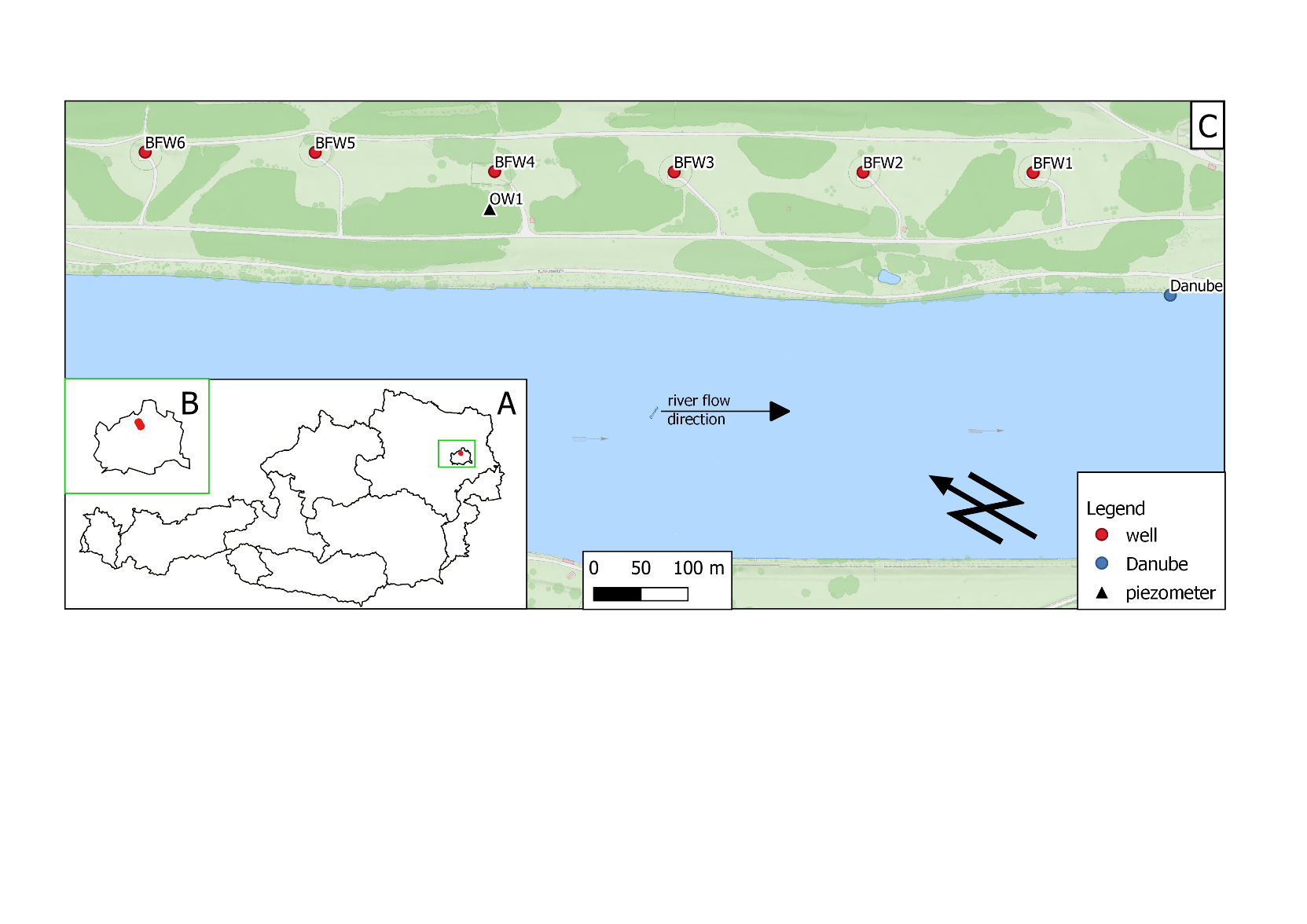
*

Fig.S 1 Map of the study site in the North of Vienna showing the location of the production wells and piezometer.


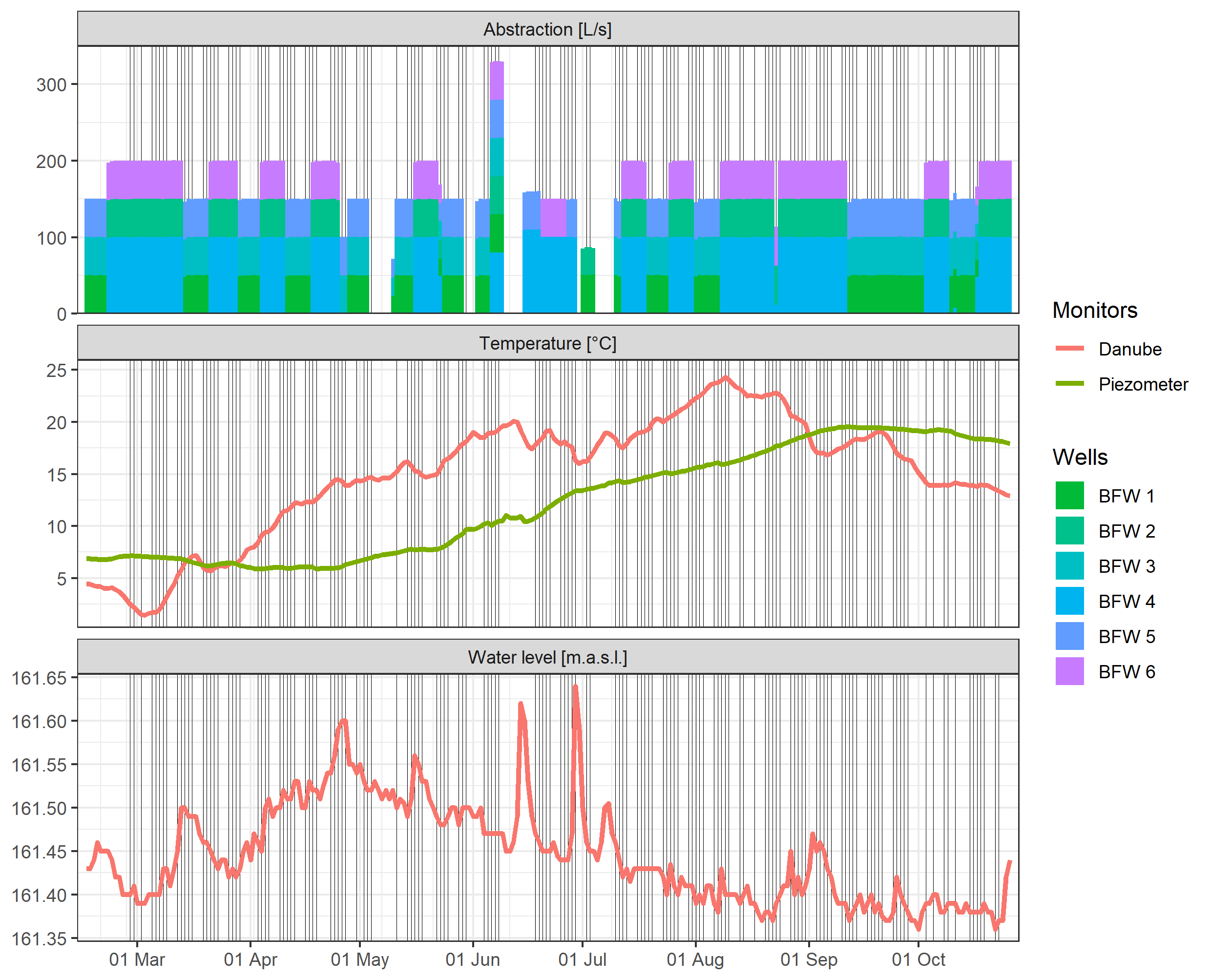


Fig.S 2 Time series of sampling (vertical black lines) with abstraction rates from the wellfield (BWF - bank filtrate wells), water temperature in Danube and Groundwater (Piezometer) and water level in Danube.

Table S 1 Substances considered in suspect screening.

Fig. S 3 shows boxplots of the relative standard deviation (RSD) of the original (uncorrected) and corrected intensities of all valid compounds from valid field samples. In the negative ionisation mode, the median of RSD is reduced from 20% to 15% by normalisation. For this ionisation mode only a inter batch correction was applied. In the positive ionisation mode, the median of RSD is reduced from 40% to 18% by normalisation. For this ionisation mode for the batches 2 and 3 additionally to the inter batch correction also drift correction was applied.


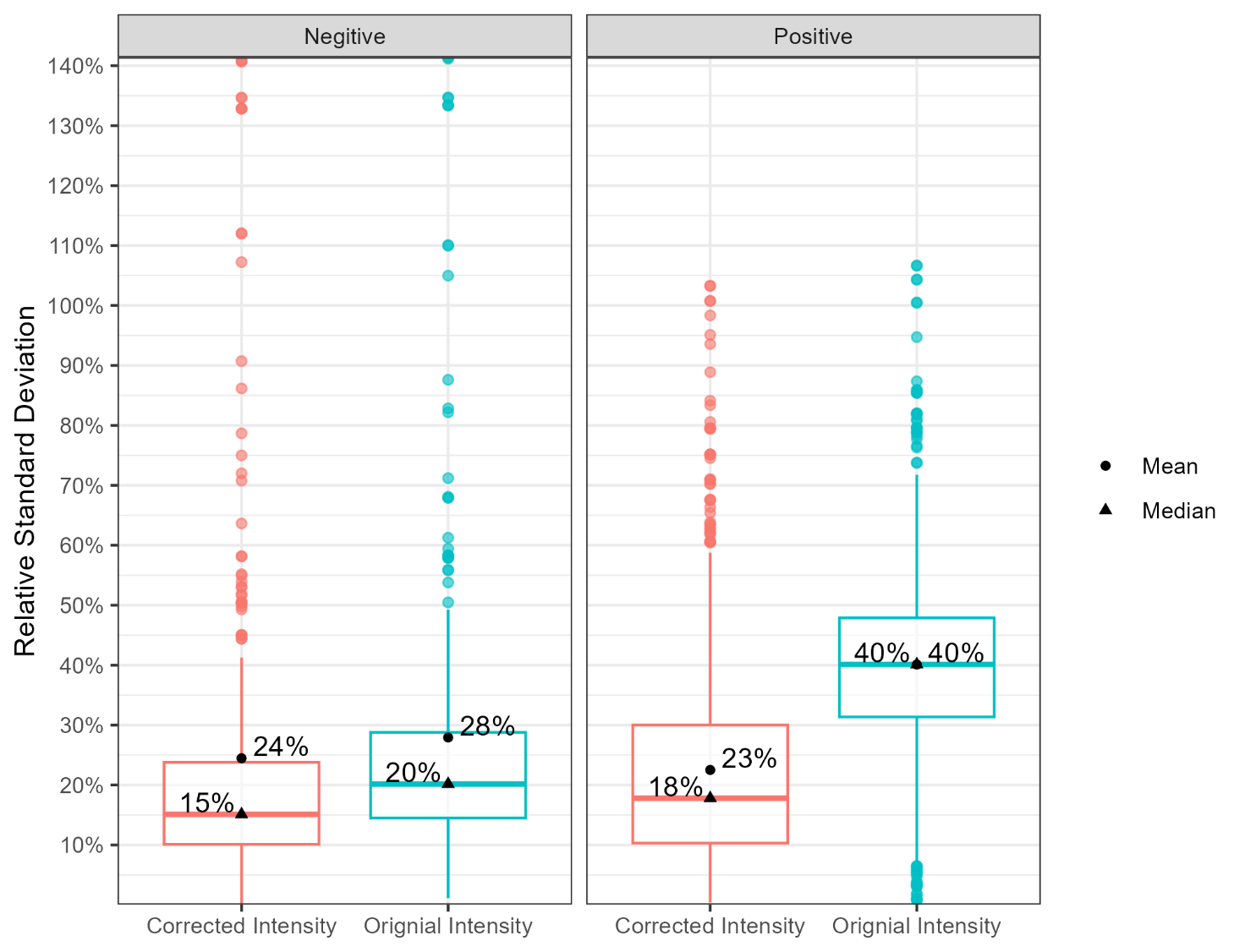


Fig.S 3 Relative standard deviation of corrected (red) and original (turquoise) intensities. Boxplot for all valid compounds in all valid samples.

Table S 2 Significance (p-value) of trend in QC samples

Table S 3 Categorisation of relative frequency of occurrence

Table S 4 Categorisation of Recurrence Dynamics

Table S 5 shows the five compounds which, considering a mass tolerance of 2 mD + 6 ppm and a retention time tolerance of 0,05 min, qualify as compounds detected with both ionisation modes.

Table S 5 Compounds qualifying for double detection

Formula S 1

$I_{mean,j}=\frac{\sum_{i=1}^{n} I_{j,i}}{n}$

$I_{mean,j}$ Mean intensity of the compound j

$I_{j,i}$ Intensity of the compound j in the sample i

$n$ Number of samples

Formula S 2

$$I_{SD,j}=\sqrt{\frac{\sum_{i=1}^{n} \left( I_{j,i}-I_{mean,j} \right)^{2}}{n-1}}$$

$I_{SD,j}$ Standard deviation of the intensity of the compound j

$I_{mean,j}$ Mean intensity of the compound j

$I_{j,i}$ Intensity of the compound j in the sample i

$n$ Number of samples


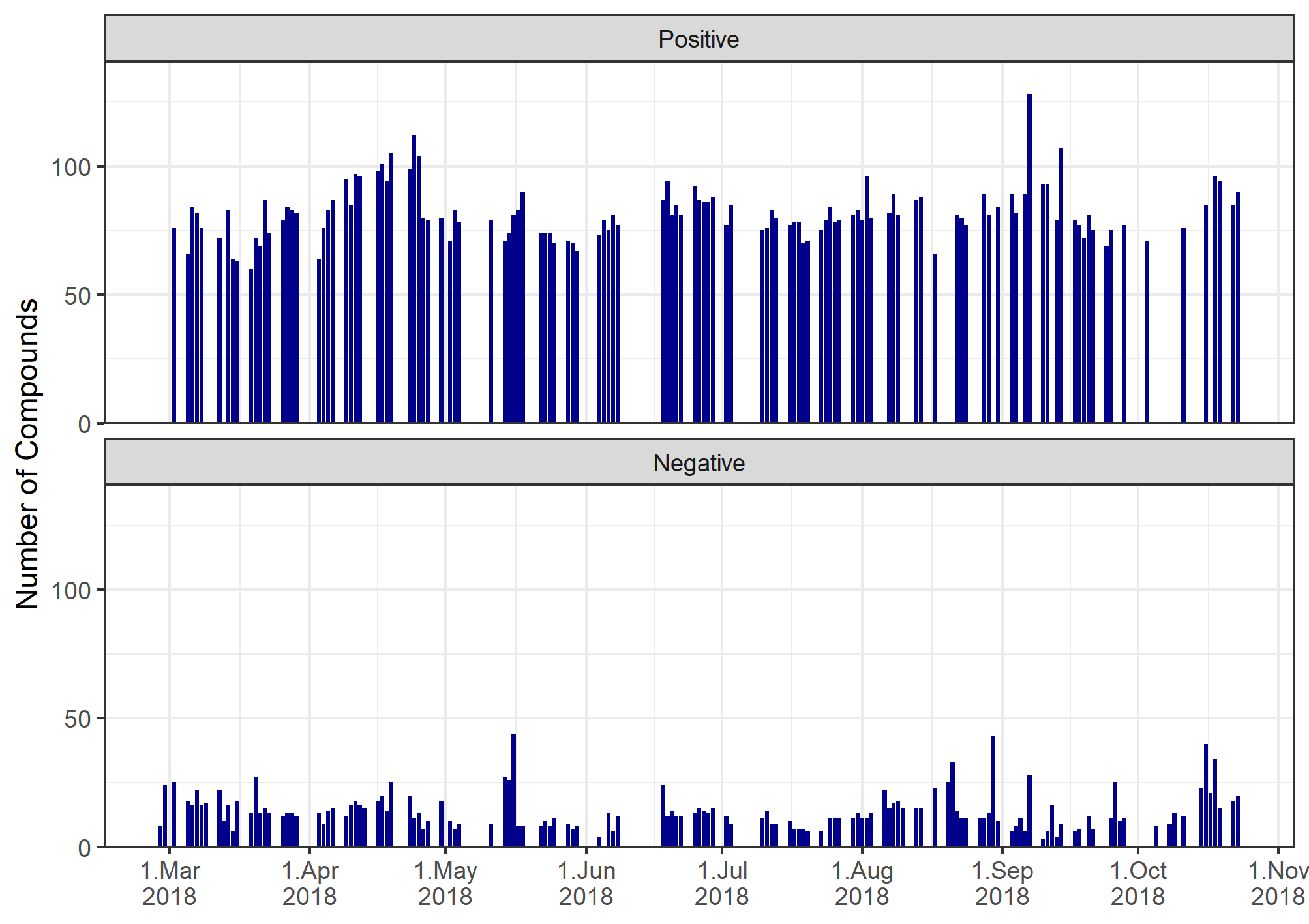


Fig.S 4 Time series of the number of compounds detected in each sample for positive (top) and negative (bottom) ionisation modes.

Fig. S 5 shows the absolute occurrence of detected compounds as well as average duration between detections for the 191 *moderately common* compounds. The more frequently a substance is detected, the further left it is shown in the diagram. The colour differentiation indicates the average temporal distance between the occurrence of the respective compound. For frequently detected compounds, the average temporal distance between detections is naturally lower than for rarely occurring ones. Compounds that occur rarely (detected ≤ 10 times) and also have a short temporal distance between each detection (magenta < 3 days) are found concentrated in a specific section of the time series, thus indicating short-term changes in the surface water. From the 67 compounds with ADBD below 3 days, 12 compounds are detected in less than 10 samples indicating concentrated occurrence.


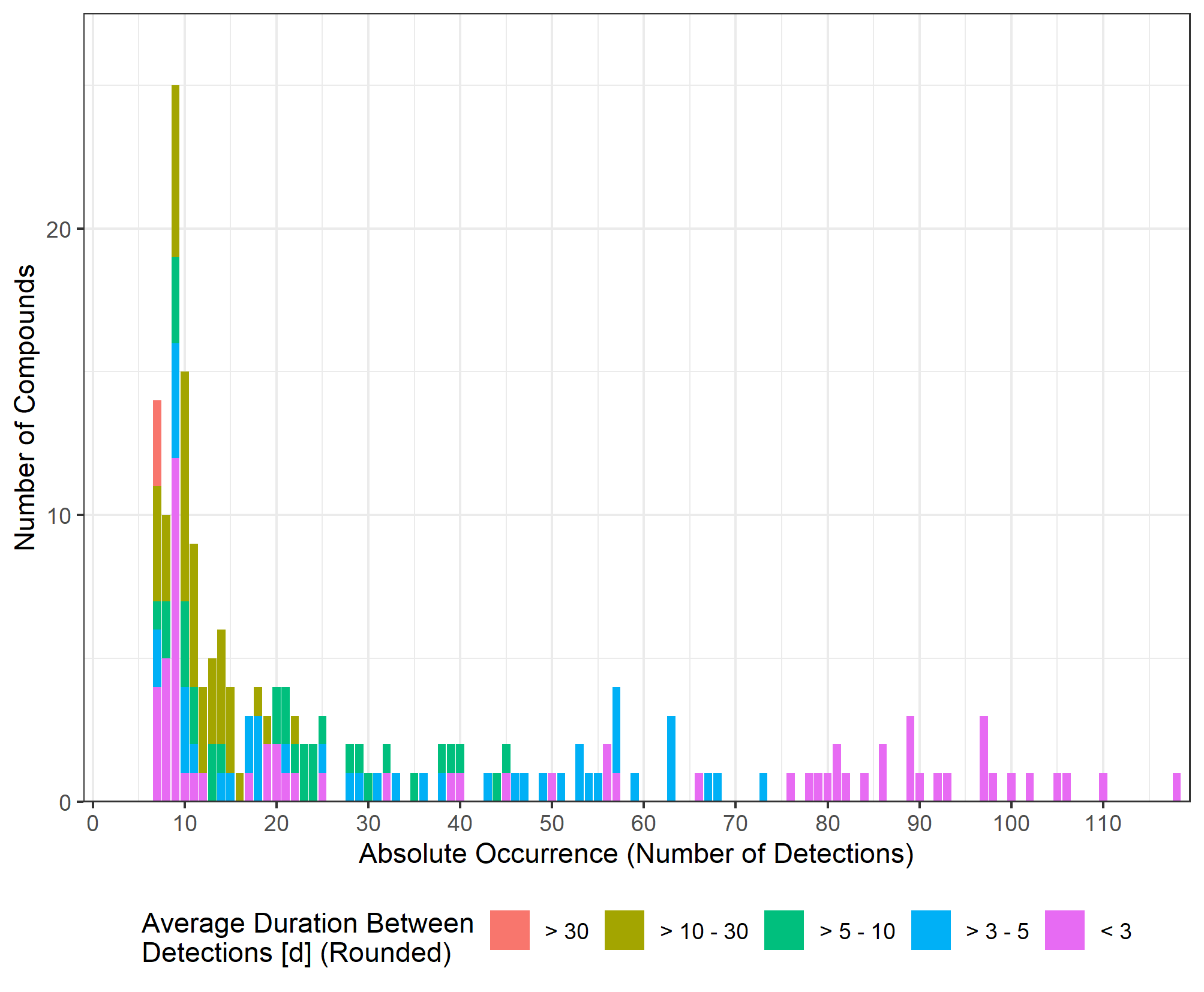


Fig.S 5 Compounds by number of detections and mean temporal distance between detections. Only moderately common compounds (relative frequency >5% and <90%) are shown.


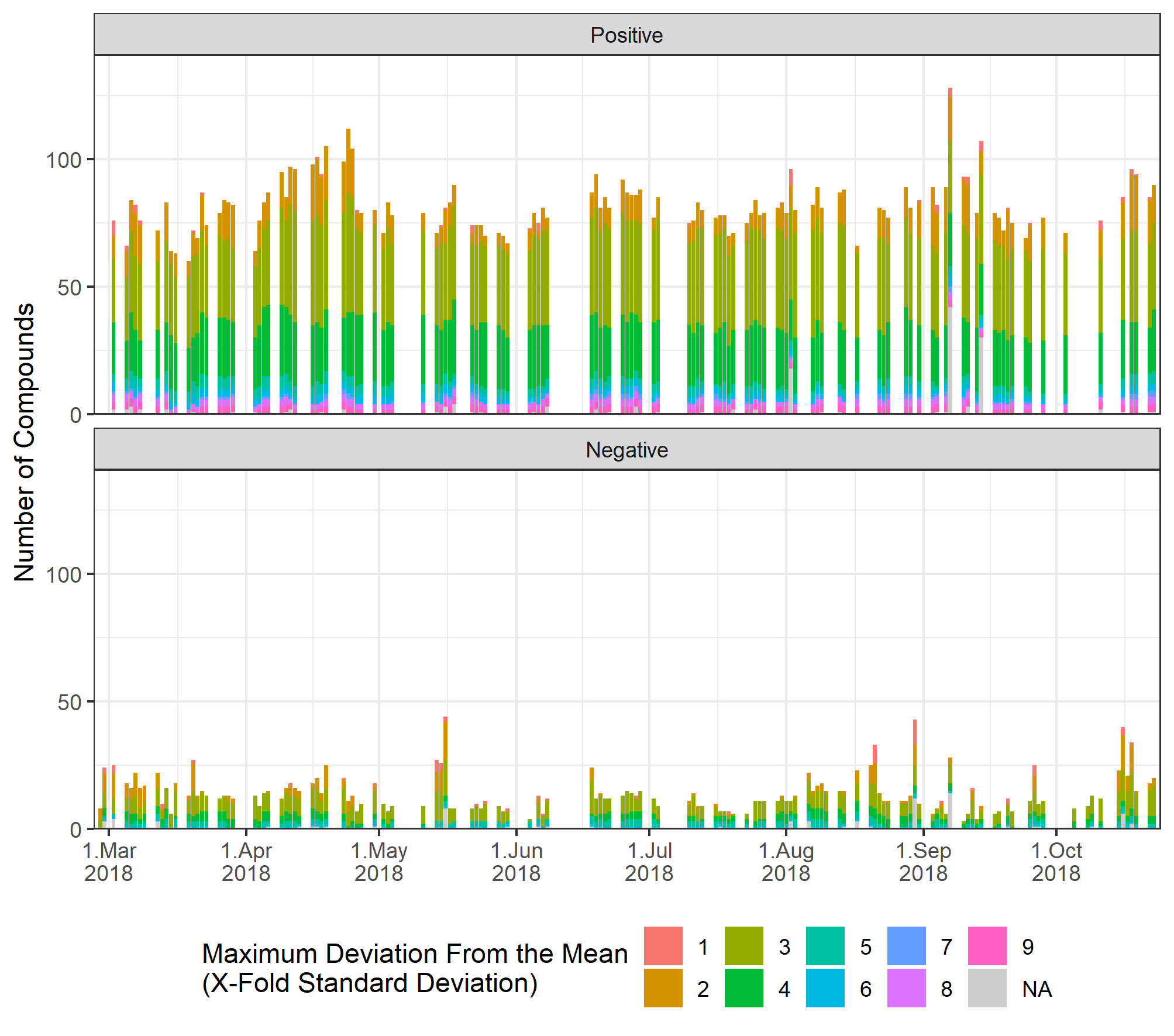


Fig.S 6 Time series of variance of intensity

Table S 6 Average share (median) of the groups with different maximum variance of intensity over all samples. (MDFM: Maximum Deviation from the Mean)

Table S 7 Evaluation of temporal dynamic and quantitative dynamic of 12 detectable substances from suspected screening

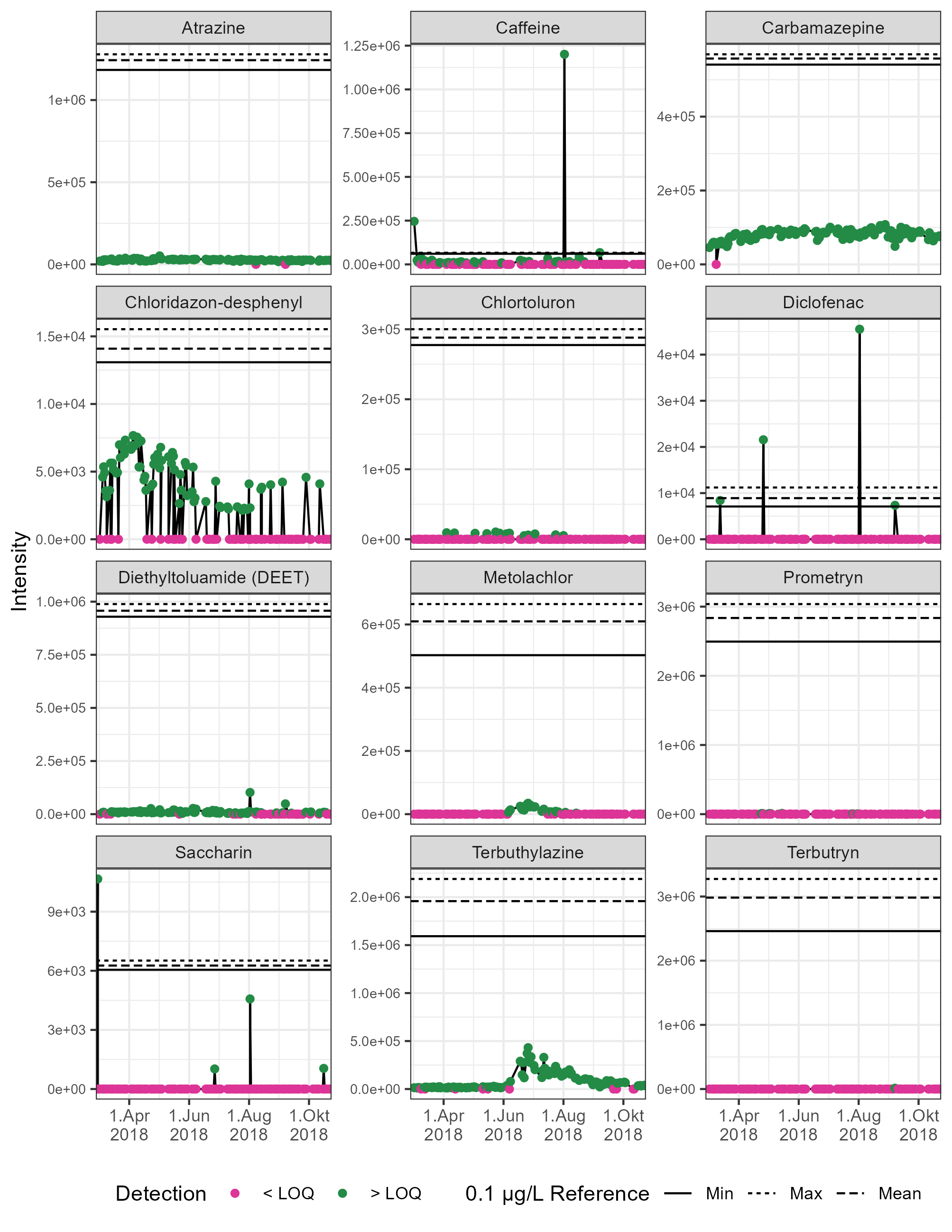


Fig.S 7 Time series of intensities of substances detected above the detection limit. The horizontal lines indicate the minimal, median, and maximal values of the intensities in the 0.1 µg/L reference samples. The measurement values in the environmental samples are shown as green dots connected by a line. Measurements that were below the detection limit are represented as pink dots at the intensity of zero.

Table S 8. Results from suspect screening (SSC) and non-targeted analysis (NTA) separated in detection classes (field samples and repetitions)
